# A comparative study on the stability of the furfural molecule on the low index Ni, Pd and Pt surfaces

**DOI:** 10.1098/rsos.211516

**Published:** 2022-03-23

**Authors:** Alveena Z. Khan, Jacob Alitt, Rhiannon Germaney, Ikutaro Hamada, Peter P. Wells, Nikolaos Dimitratos, C. Richard A. Catlow, Alberto Villa, Arunabhiram Chutia

**Affiliations:** ^1^ School of Chemistry, University of Lincoln, Brayford Pool, Lincoln LN6 7TS, UK; ^2^ Department of Precision Engineering, Graduate School of Engineering, Osaka University, 2-1 Yamadaoka, Suita, Osaka 565-0871, Japan; ^3^ UK Catalysis Hub, Research Complex at Harwell, Rutherford Appleton Laboratory, Harwell Oxon, Didcot OX11 OFA, UK; ^4^ School of Chemistry, University of Southampton, University Road Southampton, Southampton SO17 1BJ, UK; ^5^ Department of Industrial Chemistry ‘Toso Montanari’, Alma Mater Studiorum-University of Bologna, Viale Risorgimento 4, 40136 Bologna, Italy; ^6^ Department of Chemistry, University College London, Gordon Street, London WC1H 0AJ, UK; ^7^ Cardiff Catalysis Institute, School of Chemistry, Cardiff University, Cardiff CF10 3AT, UK; ^8^ Department of Chemistry, Università degli Studi di Milano, Via Golgi 19, 20133, Milano, Italy

**Keywords:** density functional theory, catalysis, furfural, stability, pristine metal surface

## Abstract

We present a comparative density functional theory investigation of the furfural (Ff) molecule on the low index Ni, Pd and Pt surfaces to understand its geometrical and electronic properties to gain mechanistic insights into the experimentally measured catalytic reactivities of these metal catalysts. We show that the number of metal *d-*states, which hybridize with the nearest C and O *p*-orbitals of the Ff molecule, can be used to explain the stability of the Ff molecule on these surfaces. We find that the hybridization between atoms with higher electronegativity and the metal *d-*states plays a crucial role in determining the stability of these systems. Furthermore, we also find electron transfer from metal to the Ff molecule on the Ni and Pd surfaces, with a reverse process occurring on the Pt surface.

## 1. Introduction

**Scheme 1.**
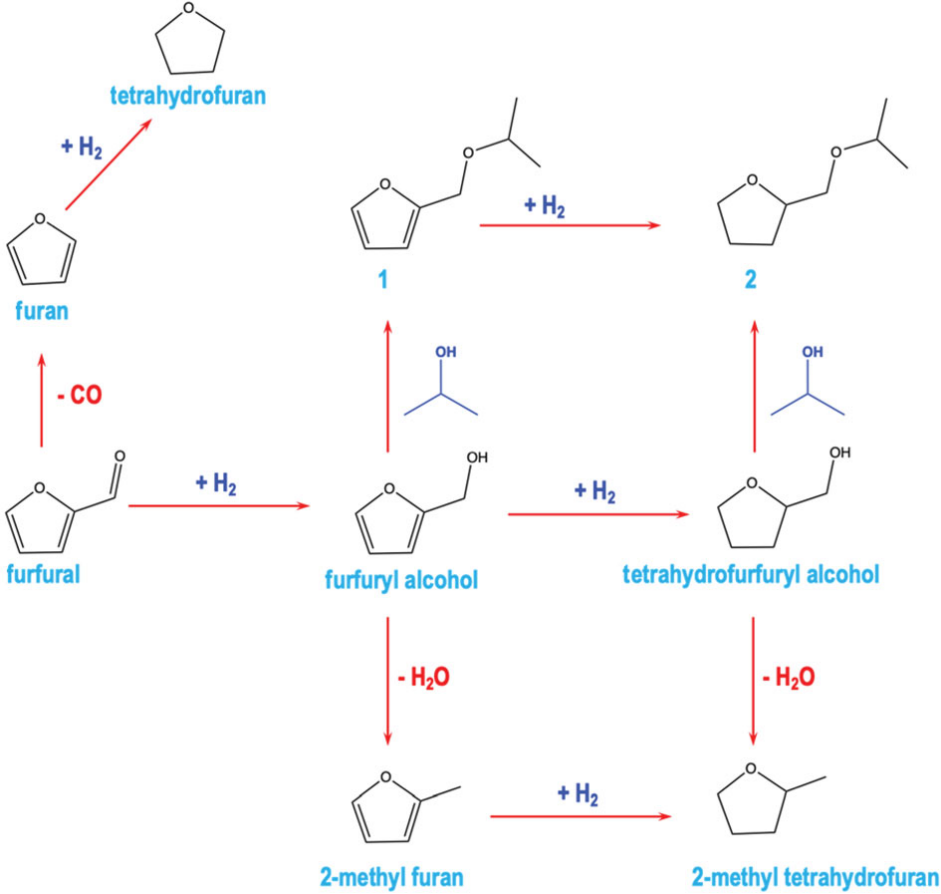
Chemical routes for furfural valorization.

Harvesting useful products from biomass and renewable feedstocks is a crucial component of the transition from a petroleum-based to a sustainable chemical industry [1–3]. In this regard, furfural (Ff) is an important precursor in the production of biofuels and many chemical intermediates [4,5]. Produced from the xylan contained in lignocellulose via hydrolysis and dehydration [6,7], hydrogenation of Ff gives intermediate chemicals with wide applications [8,9]. One such example is furfuryl alcohol (FA), which is produced from selective hydrogenation of Ff, and has a broad spectrum of applications, including the manufacture of resins, adhesives, fibres [10] and fuel derivatives, such as 1,5-pentanediols [11]. Moreover, it can undergo further hydrogenation to give tetrahydro-FA (THFA), an environmentally friendly solvent used in the automotive and agricultural industries [12,13]. Other intermediates produced from the hydrogenation of Ff include 5-hydroxymethylfurfural, 2-methyl furan and 2-methyltetrahydrofuran (see scheme 1) [1–3,14]. These intermediates are parent molecules for various useful chemicals such as caprolactone, 2,5-dimethylfuran, 2,5-furandicarboxylic acid, etc [15–17].

The nature and distribution of these intermediates is dependent on their affinity for different metal catalysts and preferential adsorption onto catalytic sites (e.g. step edges and kinks) [18,19]. A good metal catalyst should be highly active under mild conditions, withstand multiple cycles before deactivation, use economical materials and have a high selectivity for the desired product. The industrial process of gas phase hydrogenation of Ff using Cu on asbestos was first reported in 1929, and later, Du Pont de Nemours patented the use of copper chromite [20,21]. It is further reported that the Quaker Oats Company then used Cu supported on Na_2_O · SiO_2_ to achieve yields of 99% of FA, which set the precedent for many other catalytic systems based on Cu and other metals to be used in this process, providing an alternative to the use of chromites [22].

A wide range of catalysts have now been studied for the hydrogenation of Ff, with Cu-, Ni-, Ru-, Pt- and Pd-based systems being popular. Ni catalysts have been found to favour adsorption of Ff over FA, with the turn over frequency (TOF) almost constant for different average sizes of Ni metal particles, whereas in the conversion of FA to THFA, the smaller Ni particles gave higher TOF values [18]. Pushkarev *et al*. [23] reported the influence of the size of Pt nanoparticle catalysts on the production of furan and FA, where an increase of Pt nanoparticle size to 7 nm increases selectivity towards hydrogenation of Ff into FA from 1% to 66%. By contrast, the decrease of nanoparticle size was found to favour decarbonylation of Ff to furan, with 1.5 nm producing the highest selectivity (96%). Hydrogenation with SiO_2_-supported Pd-Ir bimetallic catalysts has been found to give increased TOF values with further addition of Ir as compared with Pd/SiO_2_, probably owing to the promoting effect of Ir on the adsorption at the carbonyl (C=O) site, whereas the Pd surface was found to strongly interact with the furan ring [24]. At high temperatures, a flat orientation through the furan ring and carbonyl functionality gives decarboxylation of Ff to produce furan and tetrahydrofuran [25].

Selectivity can be controlled by blocking specific sites on the catalyst surface to guide distribution, for example by using thiolates or polymers [26,27]. The use of thiolate self-assembled monolayers was found to strongly suppress Ff decarboxylation with increasing sulfur density [27]. Pang *et al.* [27] demonstrated the use of thiolates as blocking agents, leaving particle edges/corners exposed. Rogers *et al.* [26] demonstrated the use of poly(vinyl alcohol) (PVA) to direct selectivity. The nature of the PVA–Pd interactions and the relative proportion of available surface sites direct the reaction towards the hydrogenation of Ff. Jeong *et al.* [28] reported the use of Ni nanoparticle catalysts capped with organic molecules, wherein steric hinderance of the Ni surface induced by the organic layer gave selective hydrogenation of Ff to FA, with a yield of 95%. Wang *et al.* [29] recorded the use of zeolite crystals to control selectivity in the hydrogenation of Ff. The catalyst used combined the high activity of Pd nanoparticles with the selectivity of zeolite micropores to give a furan selectivity as high as 98.7%. Xue *et al*. [30] found that the optimal adsorption configuration of Ff to be the top-Ni-top-Ni site on a Ni/Pd(111) catalyst.

Periodic density functional theory (DFT) calculations have been used to explore further the optimal mechanism of Ff conversion. Ren *et al.* [31] reported the hydrodeoxygenation and decarbonylation mechanisms of Ff to proceed on the Ni(111) surface via adsorption of both the carbonyl group and the furanic ring. Despite numerous studies on the interaction of the Ff molecule on transition metal catalysts, there are considerable uncertainties on the chemical bonding and charge transfer behaviour between Ff and metal catalysts, which can be crucial in designing novel catalysts. Further to this, in one of our recent studies, we have shown the dual site hydrogenation mechanism in which the hydrogen dissociation takes place on the metal nanoparticles, and after spillover of the adsorbed hydrogen on the support material, hydrogenation of the Ff molecule takes place to yield THFA [32]. Therefore, it is important to clarify how the Ff molecule interacts with the pristine metal surfaces to optimize the catalyst. In this study, we report experimental catalytic studies, which motivated the DFT based theoretical studies with dispersion corrections to investigate the geometries, energetics and the electronic properties of the Ff molecule adsorbed on the low index Ni, Pd and Pt catalyst surfaces.

## 2. Methodologies

### 2.1. Experimental

#### 2.1.1. Catalyst preparation

*Pd catalyst*: solid Na_2_PdCl_4_ (0.051 mmol) (Sigma-Aldrich, purity greater than 99.9%) and 1 ml of a PVA solution (1 wt%) were added to 100 ml of H_2_O (Pd/PVA 1/0.5 wt/wt %). After 3 min, a 0.1 M of NaBH_4_ solution (Pd/NaBH_4_ 1/8 mol mol^−1^) was added to the solution under vigorous magnetic stirring. Within a few minutes of their generation, the colloids (acidified at pH 2, by sulfuric acid) were immobilized by adding the support (TiO_2_, Degussa P25) to the vigorously stirring solution. The amount of the support was controlled, in order to obtain a final Pd loading of 1 wt% (on the basis of quantitative loading of the metal on the support). The catalysts were filtered and washed several times and dried at 100°C for 2 h.

*Pt catalyst*: solid K_2_PtCl_4_ (0.051 mmol) (Sigma-Aldrich, purity greater than 99.9%) and 1 ml of a PVA solution (1 wt%) were added to 100 ml of H_2_O (Pt/PVA 1/0.5 wt/wt %). After 3 min, a 0.1 M of NaBH_4_ solution (Pd/NaBH_4_ 1/8 mol mol^−1^) was added to the solution under vigorous magnetic stirring. Within a few minutes of their generation, the colloids (acidified at pH 2, by sulfuric acid) were immobilized by adding the support (TiO_2_, Degussa P25) to the vigorously stirring solution. The amount of the support was controlled in order to obtain a final Pt loading of 1 wt% (on the basis of quantitative loading of the metal on the support). The catalysts were filtered and washed several times and dried at 100°C for 2 h.

*Ni catalyst*: Ni(NO_3_)_2_ · 6H_2_O (5 × 10^−4^M) and urea (urea/Ni 10:1 mol mol^−1^) were dissolved in 200 ml of water under magnetic stirring in the presence of TiO_2_ (Degussa P25). The amount of the support was controlled in order to obtain a final Ni loading of 1 wt% (on the basis of quantitative loading of the metal on the support). The solution was kept under stirring for 6 h at 80°C. The solid was separated from the solution by filtration and washed several times. The powder was dried at 60°C for 12 h and then calcined at 300°C for 3 h. Successively the catalyst was reduced under H_2_ at 300°C for 3 h.

#### 2.1.2. Catalytic activity

As noted, Ni-, Pd- and Pt-based catalysts were prepared using TiO_2_ (P25, 50 m^2^ g^−1^) as support with a loading of 1 wt%. The metal loading was confirmed by atomic absorption spectroscopy. Pd/TiO_2_ and Pt/TiO_2_ were prepared employing an established sol-immobilization method using PVA as protective agent and NaBH_4_ as reducer. Ni/TiO_2_ was prepared using a method involving the precipitation of Ni(OH)_2_ in aqueous solution, with subsequent calcination at 300 h followed by a reduction step at 300°C. Transmission electron microscopy analysis showed that Pd/TiO_2_, Pt/TiO_2_ and Ni/TiO_2_ catalysts have particle sizes of 2.7, 3.6 and 5.3 nm, respectively [32,33].

Ff (purity 99%, Sigma-Aldrich) hydrogenation was performed at 323 K, using a stainless-steel reactor (30 ml capacity), equipped with heater, mechanical stirrer, gas supply system and thermometer. Ff solution (15 ml; 0.3 M in 2-propanol) was added into the reactor, and the desired amount of catalyst (F/metal ratio = 500 wt/wt) was suspended in the solution. The pressure of the hydrogen was 5 bar.

The mixture was heated to the reaction temperature, 423 K, and mechanically stirred (1250 rpm). At the end of the reaction, the autoclave was cooled down. Samples were removed periodically (0.2 ml) for a HP 7820A gas chromatograph equipped with a capillary column HP-5 30 m × 0.32 mm, 0.25 µm Film, by Agilent Technologies. Identification of products was performed using a Thermo Scientific Trace ISQ QD Single Quadrupole GC-MS equipped with a capillary column HP-5 30 m × 0.32 mm, 0.25 µm Film, by Agilent Technologies. Authentic samples were also analysed to determine separation times. Quantitative analyses with external standard method (n-octanol) were used.

### 2.2. Computational details

The Vienna Ab-initio Simulation package (VASP) is used to perform all the DFT-based calculations [34,35]. We used the projector augmented wave method, with the plane wave cut off of 550 eV for the expansion of the wave functions, which provided bulk total energies converged to within 10^−5^ eV [36]. For the structural optimizations, we chose a convergence criterion of 0.01 eV A^−1^. For the exchange correlation functional, we used the Perdew-Burke-Ernzerhof (PBE) generalized gradient approximation [37]. A 3 × 3 × 1 Monkhurst-Pack grid was used. The ideal M(*h*,*k*,*l*) surfaces (M = Ni, Pd, Pt and *h*,*k*,*l* are 0 or 1) were modelled by seven atomic layers slab with a (4 × 4) periodicity. The slabs were constructed using the optimized lattice constants of 3.50 Å (Exp.: 3.50 Å), 3.86 Å (Exp.: 3.89 Å) and 3.93 Å (Exp.: 3.91 Å) for Ni, Pd and Pt, respectively [38]. During the optimization, the top three atomic layers were relaxed, and the bottom four layers were fixed to their respective bulk positions. Along the surface normal direction, a vacuum of approximately 15 Å was inserted, which is sufficient to eliminate spurious interactions with the neighbouring image slabs. The Ff molecule was adsorbed on one side of the slab. The dipole moment due to the adsorbed species was accounted for by using the methods of Neugebauer *et al.* [39,40]. In this study, we also employed Grimme's dispersion correction (DFT + D3), as dispersion forces might be significant for adsorption of organic molecules [41].

For the calculations of adsorption energies (E_ad_) of Ff on the M(*h*,*k*,*l*) surface, we used the following equation

2.1 $$E_{\mathrm{ad}}=E_{\mathrm{Ff}/M}-E_{M}-E_{\mathrm{Ef}},$$

where *E*_Ff/*M*_ is the total energy of the Ff adsorbed on the metal surface, *E_M_* is the total energy of the relaxed pristine surface and *E*_Ff_ is the total energy of the Ff molecule in the gas phase. To obtain *E_Ff_*_/*M*_, the adsorption of Ff was allowed on various sites on the M(*h*,*k*,*l*) surface i.e. the on-top site, in between the on-top sites and on the hollow site with respect to the O-atom of the furan ring of the Ff molecule. On the M(110) surface, the Ff molecule was allowed to adsorb along the ridges and perpendicular to ridges. The atomic charges were calculated using the Bader charge analysis as implemented by Henkelman and coworkers [42]. The VESTA package was used for visualization of the optimized structures and to measure the interatomic distances, bond angles and dihedral angles [43,44].

In addition to the periodic DFT calculations, we also use ORCA on the Ff molecule to obtain the characteristics of the highest occupied molecular orbital (HOMO) and the lowest unoccupied molecular orbital (LUMO) [45]. All the calculations were performed using the PBE functional with Grimme's D3 dispersion correction and def-2-TZVP basis set. Further to this, partial density of states (PDOS) onto the molecular orbitals and density of states weighted by the crystal orbital overlap population (COOP) were calculated using the STATE package [46–50]. In the implementation of COOP in the STATE package, the wave functions of the adsorption system are expanded in terms of the molecular orbital of the Ff molecule and the substrate wave functions. This approach is appropriate and effective for characterizing the bonding mechanism of the molecule, especially when the molecular orbitals are localized. These calculations were performed using the geometry optimized using the VASP code, and no further optimizations were performed. A *Γ*-centred 4 × 4 k-point set was used.

## 3. Results and discussion

In this section, we will present our results on the catalytic activity of Ni, Pd and Pt, the trends of the calculated adsorption energies of the Ff molecule on the M(*h*,*k*,*l*) surfaces (M = Ni, Pd and Pt), followed by discussions on the geometrical and local electronic properties of these systems.

### 3.1. Catalytic activity

The catalysts were evaluated for the hydrogenation of Ff under mild conditions (table 1); Ff 0.3 M, Ff/metal ratio 500 mol mol^−1^, 5 bar H_2_, 50°C and 2-propanol as solvent. To separate the effect of the nature of the metal and particle size effect, the activity was calculated based on the superficial atoms using a procedure reported previously (table 1) [33].

**Table 1.** Furfural hydrogenation in 2 propanol.

catalyst^a^	activity^b^	selectivity (%)					
		furfuryl alcohol	tetrahydrofurfuryl alcohol	2-methyl furan	tetra hydro methyl furan	isopropyl furfuryl ether	isopropyl tetrahydro furfuryl ether
Ni/TiO_2_	261	14	81	1	—	—	3
Pd/TiO_2_	981	69	24	—	—	4	1
Pt/TiO_2_	1018	85	8	—	—	6	—

^a^Reaction conditions: furfural = 0.3 M; F/metal ratio = 500 wt/wt, 323 K, **5 bar H_2_**.

^b^Mol of furfural converted per hour per surface atoms of metal, calculated after 15 min of reaction.

The catalytic activity demonstrated that the 1 wt% Pt/TiO_2_ catalyst (1018 h^−1^) and Pd/TiO_2_ (981 h^−1^) exhibited superior activity as compared to the Ni/TiO_2_ (261 h^−1^) catalyst. Notably, not only was there a difference in activity, but there was also a pronounced change in the selectivity profile. The Ni/TiO_2_ catalyst had a preferential selectivity towards THFA (81%), whereas Pd/TiO_2_ and Pt/TiO_2_ favoured FA (69% and 85%, respectively). This observation is consistent with studies that have already proved that Ni is able to hydrogenate both the carbonyl group and furanic ring [32]. To understand the reasons behind the observed catalytic activities of the Pt/TiO_2_, Pd/TiO_2_ and Ni/TiO_2_ catalyst, studies on the adsorption of the Ff molecule on these catalysts followed by the mechanism of Ff hydrogenation are required. As a first step towards addressing the detailed reaction mechanism, we focus on theoretical understanding of the nature of the interaction between the Ff molecule and the low index Ni, Pd and Pt surfaces in the following sections.

### 3.2. Adsorption energies

**Figure 1.**
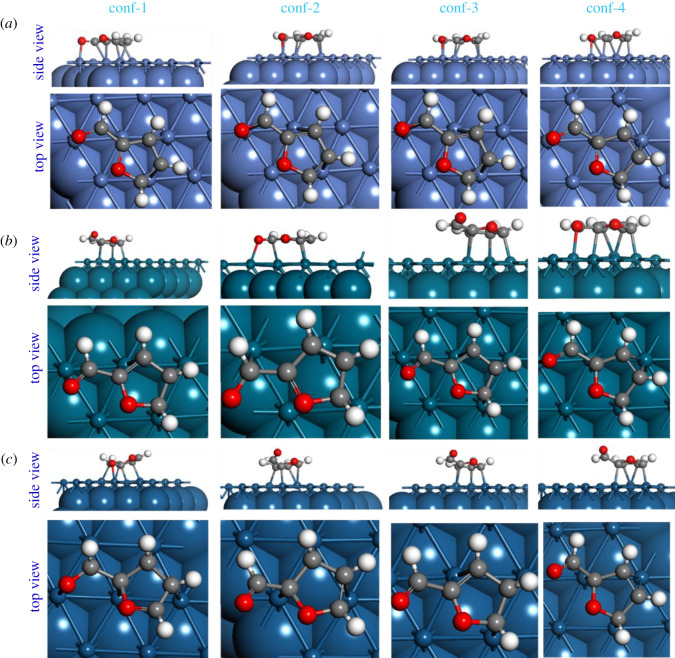
The fully optimized structures of the four configurations of the Ff molecule on the (*a*) Ni(111), (*b*) Pd(111) and (*c*) Pt(111) surfaces. For clarity, the exposed surface including the adsorbed molecule is shown in ball and stick form, and the rest of the model is represented in the CPK form.

**Figure 2.**
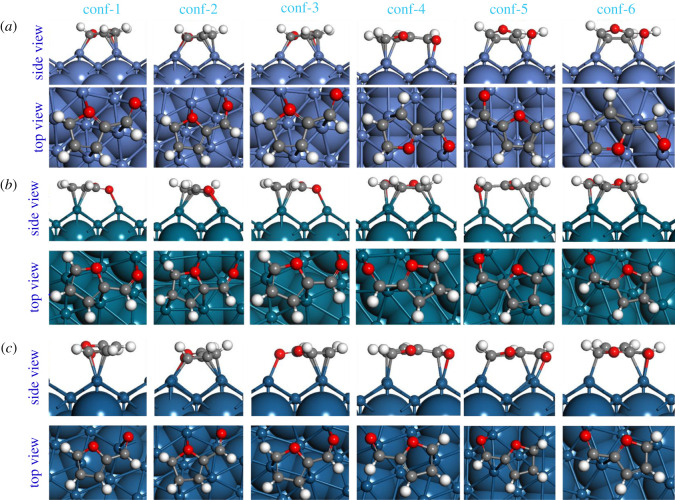
The fully relaxed structures of the six different configurations of the Ff molecule on the (*a*) Ni(110), (*b*) Pd(110) and (*c*) Pt(110) surfaces. For clarity, the exposed surface including the adsorbed molecule is shown in ball and stick form, and the rest of the model is represented in the CPK form.

**Figure 3.**
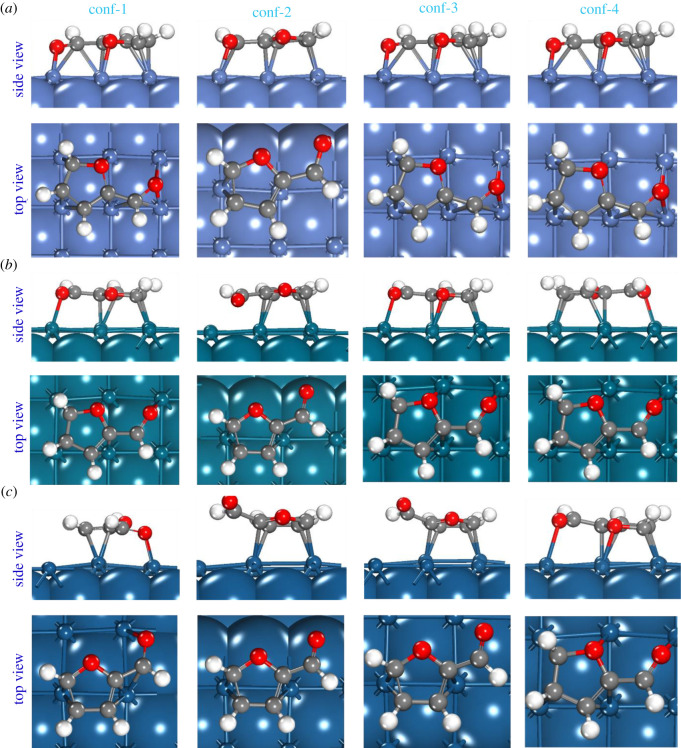
The fully optimized structures of the four configurations of the Ff molecule on the (*a*) Ni(100), (*b*) Pd(100) and (*c*) Pt(100) surfaces. For clarity, the exposed surface including the adsorbed molecule is shown in ball and stick form, and the rest of the model is represented in the CPK form.

The calculated adsorption energies of the Ff molecule on the low index Ni, Pd and Pt surfaces are summarized in table 2. For uniformity, similar initial configurations of the Ff molecule on all these surfaces were generated, and the adsorption energies are calculated on the fully relaxed Ff/M(*h*,*k*,*l*) systems. Previously, we have shown that the parallel configuration of Ff on the catalyst surface is more stable as compared to the perpendicular configuration [26]. Therefore, only the parallel configurations at different positions on the M(*h,k,l*) surfaces are considered in this study. For clarity, all the optimized structures of various configurations used for this study are presented in figures 1–3 and the coordinates are provided in the electronic supplementary materials.

**Table 2.** The adsorption energy of furfural on the low index Ni, Pd and Pt surfaces.

configuration	adsorption energy (eV)		
	Ni	Pd	Pt
M(111)			
1	−1.83	−1.55	−1.85
2	−1.68	−1.50	−2.03
3	−1.69	−1.55	−2.03
4	−1.70	−1.58	−2.05
M(110)			
1	−2.72	−2.19	−1.69
2	−2.57	−2.01	−2.62
3	−2.72	−2.19	−2.89
4	−2.34	−2.17	−2.01
5	−2.53	−2.15	−2.57
6	−2.53	−2.17	−2.63
M(100)			
1	−2.67	−2.11	−2.19
2	−2.19	−1.84	−2.21
3	−2.67	−2.11	−2.21
4	−2.67	−2.11	−2.53

On the M(111) surface, the adsorption energy of Ff is greatest (most negative) on the Pt(111) surface, followed by the Ni(111) and the Pd(111) surface (table 2). We also see that the difference between the highest and lowest adsorption energies is only 0.08 eV on the Pd(111) surface, while it is 0.15 and 0.20 eV for the Ni(111) and the Pt(111) surfaces, respectively, that is, the adsorption energies on different adsorption sites are comparable for Pd (111), while the differences of the adsorption energies on different sites are more significant for Ni(111) and the Pt(111). On the fully relaxed (110) surfaces, the adsorption energy of the Ff molecule follows a similar trend to the (111) surfaces, i.e. adsorption is most exothermic on the Pt(110) surface, followed by Ni(110) and Pd(110). It is interesting to note that unlike the (111) surfaces, adsorption energies for Pt(110) and Ni(110) are comparable. For the (100) surfaces, however, the Ff molecule is adsorbed most strongly on the Ni surface, which is followed by Pt and Pd. To clearly understand the probable reasons for the differences in the adsorption energies, we explore the geometrical and the local electronic properties of the Ff/M(*h*,*k*,*l*) systems.

### 3.3. Geometrical properties

In this subsection, we present a comparison of the geometries of the adsorbed Ff molecule with respect to the gas phase Ff molecule. For clarity, we will only consider the most stable structures of the Ff/M(*h*,*k*,*l*) systems. In table 3, the interatomic distances and the dihedral angles of the fully relaxed Ff molecules are summarized. Upon adsorption, the Ff molecule is distorted: the interatomic distances increase and the planarity (measured by the dihedral angles) of the molecules change significantly. However, there is no clear correlation between the overall molecular distortion and the calculated adsorption energies. For example, on the Ni(111) and Pd(111), both carbon atoms of the furan ring and the O-atom of the -CHO group directly interact with the surfaces (see figure 1). However, on the Pt(111) surface, the Ff molecule interacts via the C-atoms of the furan ring only, which consistent with previous studies [31,51]. Similarly, on the M(110) and M(100), the Ff molecule adsorbs via both the C-atoms of the furan ring and the O-atom of the -CHO group (see figure 2). On the (100) surfaces, the adsorption of Ff follows the same trend as on the M(111) surfaces (see figure 3). The distances between the Ff molecule and the surface are also comparable for all the systems. Based on these analyses, we conclude that the geometry of the Ff molecule may change significantly, and the mode of adsorption also varies with the metal catalysts. For example, on the most stable Ff/Ni(111) system, all the C-atoms of the furan ring and the atoms of the -CHO group are close to the Ni(111) surface (unlike the most stable Ff/Pd(111) and Ff/Pt(111) systems), which may indicate the reason for preferential selectivity of Ni towards THFA as reported in the experimental section above. However, to understand the underlying reasons for different stability of the Ff molecule on these metal surfaces, a more detailed investigation on the electronic properties is required.

**Table 3.** The interatomic distances and dihedral angles of the most stable Ff/*M*(*h*,*k*,*l*) systems under investigation. (Please refer to table 2 for the most stable configurations.)

system	interatomic distance (Å)						dihedral angles (°)					
	C−C_ring_	C−O_ring_	C−C_CHO_	C=O	Ff-M	M−M	CCCC_ring_	COCC_ring_	HCCH_ring_	OCCH_ring_	OCCO	OCCH_CHO_
furfural	1.392	1.369	1.452	1.227	—	—	0.019	0.009	0.035	180.000	0.060	180.000
M(111)												
Ni(111)	1.450	1.408	1.438	1.311	2.086	2.540	0.984	14.048	7.213	143.873	5.714	157.049
Pd(111)	1.447	1.405	1.454	1.270	2.228	2.772	1.147	19.231	10.149	149.353	13.201	13.201
Pt(111)	1.472	1.414	1.500	1.219	2.164	2.840	1.068	28.045	6.250	138.486	11.329	169.245
M(110)												
Ni(110)	1.444	1.438	1.440	1.319	2.061	2.489	1.172	8.969	42.805	155.421	5.081	159.533
Pd(110)	1.434	1.390	1.432	1.268	2.220	2.769	1.189	6.086	6.578	158.912	10.959	10.959
Pt(110)	1.454	1.392	1.437	1.268	2.191	2.788	0.634	8.059	6.155	149.724	18.736	161.041
M(100)												
Ni(100)	1.446	1.438	1.441	1.350	2.090	2.527	1.861	9.215	13.118	152.358	10.640	144.483
Pd(100)	1.449	1.422	1.458	1.274	2.206	2.804	0.634	7.193	15.297	149.782	9.647	160.868
Pt(100)	1.452	1.395	1.486	1.220	2.179	2.875	1.426	8.878	1.350	144.873	10.077	169.951

### 3.4. Bader charges

The calculated Bader charges are summarized in table 4. To understand fully the charge distribution on the adsorbed Ff molecule, we begin with that in the gas phase. As shown in table 4, we not only consider the total charge on the Ff molecule but also those on the furan ring, furan ring skeleton (without the H-atoms) and the -CHO group, which is considered, as in most of the adsorbed configurations, the C-atoms of the furan ring are close to the surface. From the charge analysis in the gas phase, we find that the Ff molecule has both positive and negative regions i.e. the furan ring is slightly positive and the -CHO group is slightly negative. Based on the charge redistribution in these regions, we aim to understand the charge transfer phenomenon between the Ff molecule and the M(*h*,*k*,*l*) surface.

**Table 4.** The average charge on all the metal atoms of the exposed surface (M_srf_), metal atoms on the exposed surface directly interacting with the Ff molecule (M_int_), adsorbed Ff molecule (furfural), furan ring, furan ring skeleton (without the H-atoms) and the aldehyde group of the Ff molecule (CHO group).

furfural on	configuration (table 2)	average charge (e) on					
		M_srf_	M_int_	furfural	furan ring	furan ring skeleton	CHO group
Ni(111)	1	0.023	0.093	−0.063	−0.047	−0.153	−0.107
Ni(110)	3	0.040	0.158	−0.065	−0.046	−0.152	−0.115
Ni(100)	3	0.048	0.151	−0.074	−0.223	−0.404	0.332
Pd(111)	4	−0.006	0.075	−0.015	−0.007	−0.095	−0.039
Pd(110)	3	−0.007	0.091	−0.016	−0.012	−0.096	−0.046
Pd(100)	3	0.005	0.066	−0.026	−0.014	−0.093	−0.058
Pt(111)	3	−0.034	0.035	0.006	0.011	−0.066	−0.008
Pt(110)	3	−0.026	0.112	−0.007	0.004	−0.069	−0.039
Pt(100)	3	−0.040	0.059	0.006	0.009	−0.077	−0.002
furfural molecule in gas phase		—	—	0.000	0.005	0.174	−0.012

#### 3.4.1. Furfural on Ni surfaces

The investigation of the charges on the adsorbed Ff molecule on the Ni surfaces show that the overall charge on the Ff molecule is in the range of −0.06 to −0.07 e. Similarly, the charge on the furan ring and furan ring skeleton is also negative. Furthermore, when we analyse the charges of the surface atom interacting directly with the Ff molecule, we find that it is slightly positive. Clearly, there is charge transfer from the Ni surfaces to the Ff molecule (table 4). We note that in the Ff/Ni(100) system, even though the overall charge on the Ff ring is comparable to the other two systems, the charge on the furan ring, and the furan ring skeleton is more negative and the -CHO group is positive, which is presumably related to the need to facilitate a stronger interaction between the Ff molecule and the Ni(100) surface intra-molecular by a charge redistribution in the Ff molecule. From the charge analysis, it is clear that slightly positively charged Ni surfaces, and the slightly negatively charged Ff molecule leads to favourable adsorption energies.

#### 3.4.2. Furfural on Pd and Pt surfaces

In the Ff/Pd(*h*,*k*,*l*) systems, the directly interacting Pd atoms are positively charged, and the Ff molecule is negatively charged (table 4). Therefore, there is charge transfer from the Pd surface to the Ff molecule and the Pd(*h*,*k*,*l*).

On the Pt(*h*,*k*,*l*) surfaces, we find an interesting distribution of the charges on the Pt surfaces i.e. the Pt atoms bound to the Ff molecule are positively charged, and the average charge on the furan skeleton is negative, clearly indicating that there is strong interaction between the Ff molecule and the Pt surfaces. However, the average charge on the Pt surface is slightly negative which indicates net charge transfer from the Ff molecule to the Pt surface (table 4). The negative charge on the furan ring skeleton and overall, near neutrality of charge on the Ff molecule upon adsorption on the Pt surface may be owing to charge compensation from the -CHO group. From the Bader charge analysis, we understand that on the Ni and Pd surfaces, there is charge transfer from the metal to the Ff molecule. On the other hand, on the Pt surface, there may be charge transfer from the Ff molecule to the metal surface).

### 3.5. Charge density difference

**Figure 4.**
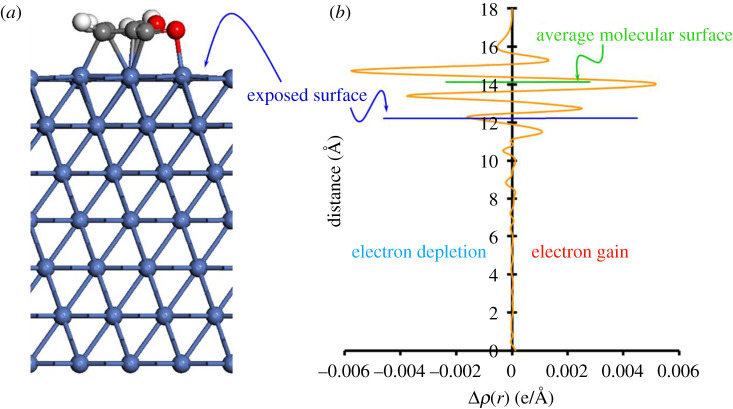
(*a*) Fully relaxed structure of the Ff/Ni(111) system and (*b*) planar average charge density difference showing accumulation and depletion of electrons above and below 0 e/Å respectively. The blue line denotes the exposed surface and the green line denotes the average molecular surface.

**Figure 5.**
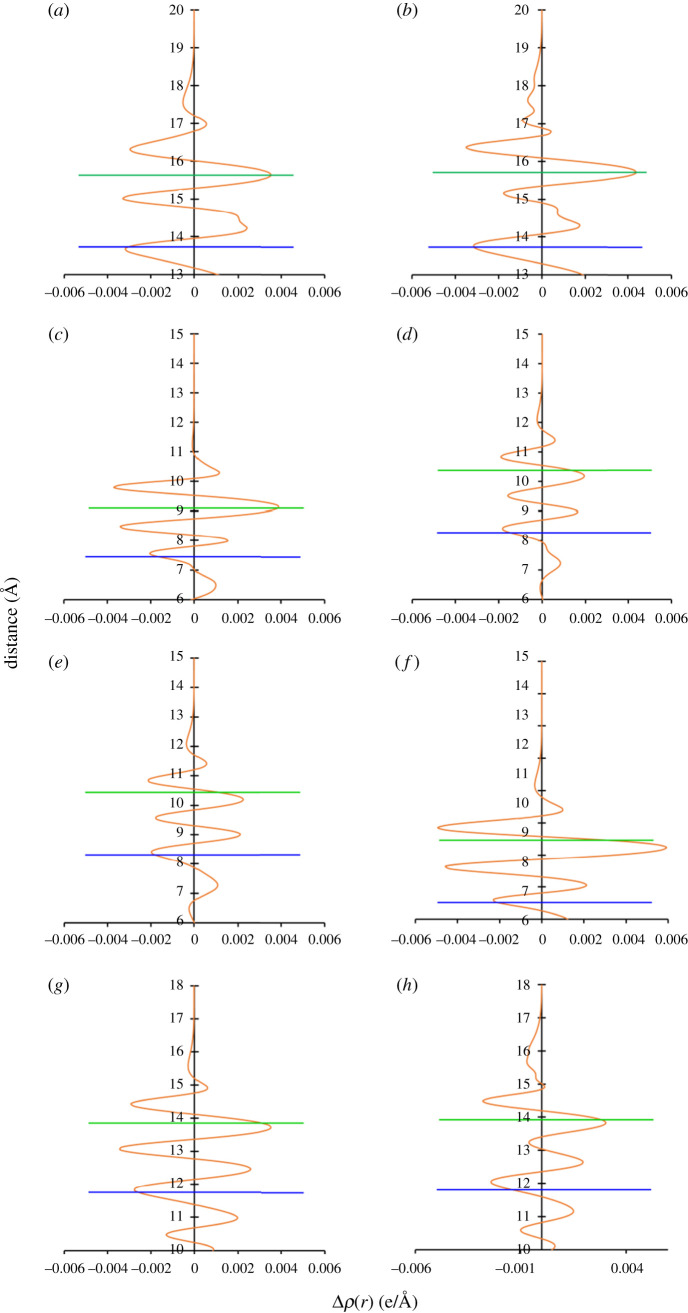
The planar average charge density difference showing accumulation and depletion of electrons above and below 0 e/Å respectively for the (*a*) Ff/Pd(111), (*b*) Ff/Pt(111), (*c*) Ff/Ni(110), (*d*) Ff/Pd(110), (*e*) Ff/Pt(110), (*f*) Ff/Ni(100), (*g*) Ff/Pd(100) and (*h*) Ff/Pt(100) surfaces. The horizontal blue and green lines represent the exposed metal surface and average molecular surface, respectively.

In this section, we present the planar average charge density difference, which is defined by:

3.1 $$\Delta \bar{\rho }(z)=\frac{1}{A}\int \int dxdy\;\Delta \rho (r),$$

where *A* is the area of the surface unit cell under consideration and Δρ(r) is the charge density difference defined by:

3.2 $$\Delta \rho (r)=\rho_{\mathrm{Ff}/M}(r)-[\rho_{M}(r)+\rho_{\mathrm{Ff}}(r)],$$

where, $\rho_{\mathrm{Ff}/M}(r)$ is the charge density of the total system, which includes the substrate and the adsorbate, and $\rho_{M}(r)$ and $\rho_{\mathrm{Ff}}(r)$ are the charge densities of the pristine surface and the isolated molecule, respectively. Figure 4 shows the fully relaxed Ff/Ni(111) system, along with the planar average charge density. We can see the electron depletion on the surface and electron gain on the Ff molecule, agreeing well with the Bader charge analysis in §3.4. We performed similar analysis on all the other surfaces, and the results are compiled in figure 5, and for clarity, the exposed metal surface and the average molecular surfaces are denoted by red and green lines, respectively. As expected, our analysis shows electron depletion on all the exposed surfaces of Ni and Pd. The $\Delta \bar{\rho }(z)$ for the Pt surfaces also display electron depletion even though the exposed surfaces have traces of negative charge on it (table 4). This can be explained by the fact that the directly interacting Pt atoms just below the Ff molecule in the Ff/Pt(*h*,*k*,*l*) system are positively charged. In addition, it also shows that there is a strong chemical bonding between the Ff molecule and M(*h,k,l*) surfaces, which agrees with the calculated adsorption energies which are in the range of −1.50 to −2.89 eV. While our study on Bader charges and planar average charge density difference provide important information on charge transfer and chemical bonding, they do not clarify the reasons for the trends in the calculated adsorption energy in the Ff/Ni(*h*,*k*,*l*) systems. Therefore, in the next step, we investigate the PDOS to understand the probable reasons for varying stability of the Ff molecule on the M(*h*,*k*,*l*) surfaces.

### 3.6. Partial density of states and chemical bonding

In this section, we systematically explore the PDOS to understand the trends of the calculated stabilities of the Ff molecules on the metal surfaces. Before presenting the detailed results, we note that in all the Ff/M(*h*,*k*,*l*) systems, one of the carbon atoms next to the O-atom (C_Oxygen_) of the furan ring has the shortest interatomic distance from the M(*h*,*k*,*l*) surface, which is related to the fact that owing to the higher electronegativity of the O-atom, there is electron depletion on this site favouring bond formation with the metal atoms underneath. In some of the systems, the O-atom of the -CHO group (O_CHO_) is also close to the metal surface. Therefore, in our PDOS analysis, we consider the PDOS of these atoms with the nearest metal atoms.

#### 3.6.1. Stability of furfural on the M(111) surfaces

**Figure 6.**
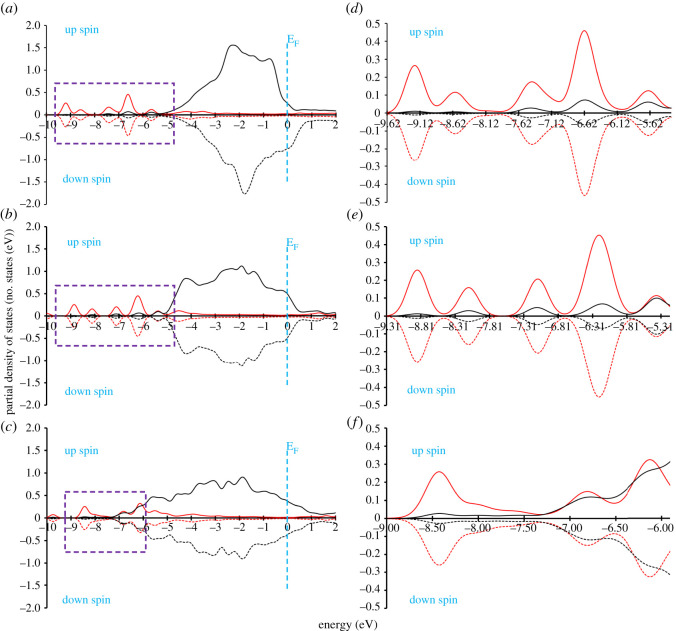
Partial density of states of the nearest carbon atom of furfural to the (*a*) Ni(111), (*b*) Pd(111) and (*c*) Pt(111) surfaces. The area under investigation (denoted by dotted rectangle) for understanding chemical bonding of the Ff molecule on the Ni(111), Pd(111) and the Pt(111) surfaces is shown in (*d*), (*e*) and (*f*), respectively. Red and black lines represent C *p* and M *d-*orbitals respectively.

First, we consider the interaction of the C_Oxygen_ atom with the nearest metal atom for the Ni(111), the Pd(111) and the Pt(111) surfaces. As shown in figure 6*a–c*, we find that upon adsorption of the Ff molecule, the C *p*-states overlap with the surface metal *d*-orbitals at the lower energy levels. For clarity, this feature is shown using a dotted rectangular box in figure 6*a–c*. To understand further this effect, we closely monitored this area (as shown in figure 6*d–f*). In the Ff/Pt(111) system, the overlapping region between the C *p-* and Pt *d*-orbitals is in the region between −9.00 and −5.91 eV. In the case of the Ff/Ni(111) and the Ff/Pd(111) system, the major overlapping region is between −9.62 and −5.31 eV and −9.31 to −5.18 eV respectively. To capture a clear picture of the overlapping region, we calculated the number of states of metal *d*-orbitals, which hybridize with the C *p*-orbital [52]. As shown in table 5, the number of states for the corresponding Pt *d*-states is 0.23, which is larger than those in the Ff/Ni(111) and the Ff/Pd(111) systems, suggesting that the chemical bonding between the Ff molecule and the Pt(111) surface is stronger than those for the other systems, leading to the most negative adsorption energy of the Ff/Pt(111) system and hence higher stability.

**Table 5.** The area under C *p* and O *p* orbitals (in number of states) in the major overlapping region.

furfural on	area of d (number of states)			
	under C		under O	
	up	down	up	down
Ni(111)	0.080	0.071	0.099	0.075
Pd(111)	0.100	0.099	0.063	0.063
Pt(111)	0.230	0.230	—	—
Ni(110)	0.058	0.050	0.103	0.084
Pd(110)	0.111	0.111	0.062	0.062
Pt(110)	0.136	0.136	0.136	0.136
Ni(100)	0.071	0.059	0.296	0.306
Pd(100)	0.076	0.076	0.070	0.070
Pt(100)	0.176	0.176	—	—

**Figure 7.**
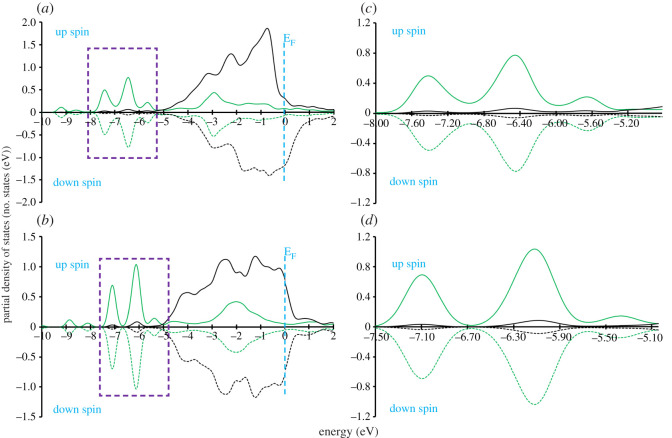
Partial density of states of the O-atom of the -CHO group of furfural to the (*a*) Ni(111) and (*b*) the Pd(111) surfaces. The area under investigation (denoted by dotted rectangle) for understanding chemical bonding of the Ff molecule on the Ni(111) and the Pd(111) surfaces is shown in (*c*) and (*d*), respectively. The green and black lines represent O *p* and M *d*-orbitals respectively.

We further saw that the number of states for the *d*-orbitals which hybridize with the C *p*-orbitals for Ff/Pd(111) systems is larger than the Ff/Ni(111), which, however, does not explain the trend of the calculated adsorption energy. As we mentioned earlier, the Ff molecule in the Ni(111) and the Pd(111) systems interact via the O of the -CHO group as well. Therefore, as shown in figure 7, the consideration of the number of states for the metal *d*-orbitals which hybridize with the O *p*-orbitals is also required. As summarized in table 5, the number of states for the metal *d*-orbitals which hybridize with the O *p*-orbitals for the Ni(111) system is larger than that for the Pd(111) system. From this analysis, we conclude that the atoms with higher electronegativity (in this case, O as compared to C) play a key role in determining higher stability of the interacting systems.

#### 3.6.2. Stability of furfural on the M(110) and the M(100) surfaces

In the case of the Ff/M(110) systems, both C- and O-atoms of the Ff molecule are within the bonding regions of the metal surfaces. Therefore, as explained in the previous section, the number of states for the metal *d*-orbitals which hybridize with the O *p*-orbitals is considered. The results show that the number of states for the Ff/Pt(110) system is larger than Ni(110), followed by Pd(110), indicating that the Ff molecule is chemically bonded more strongly to the Pt(110) surface followed by Ni(110) and Pd(110), in line with the calculated adsorption energies shown in table 2. Using the same approach for the Ff/M(100) systems, we find that the Ff/Ni(100) system has the largest number of states for the hybridizing *d*-orbitals (table 5), enabling the Ff molecule to bond strongly to the Ni(100) surface, followed by Pt(100) and Pd(100) surfaces. Since there is no direct interaction between the O-atoms of the Ff molecule with the Pt(100) surface, the number of *d-*orbitals hybridizing with the C *p*-orbitals is considered. The PDOS analysis for the *d*-orbitals of the surface metal and *p*-orbitals of the interacting C- and O-atoms allows a more detailed understanding of the trends in the calculated adsorption energy.

### 3.7. Crystal orbital overlap population

**Figure 8.**
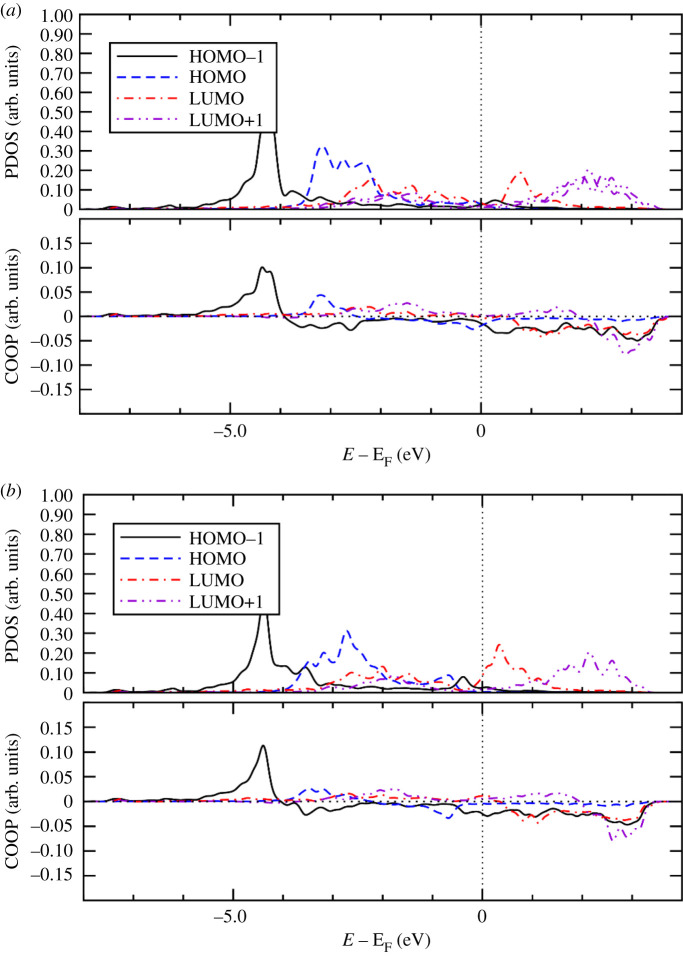
Density of states projected onto molecular orbitals (PDOS) and density of states weighted by crystal orbital overlap population (COOP) for the Ff molecule adsorbed on Ni(110). (*a*) spin up, (*b*) spin down. Gaussian broadening with the width of 0.01 eV was used. Origin of the energy is the Fermi level (E_F_).

**Figure 9.**
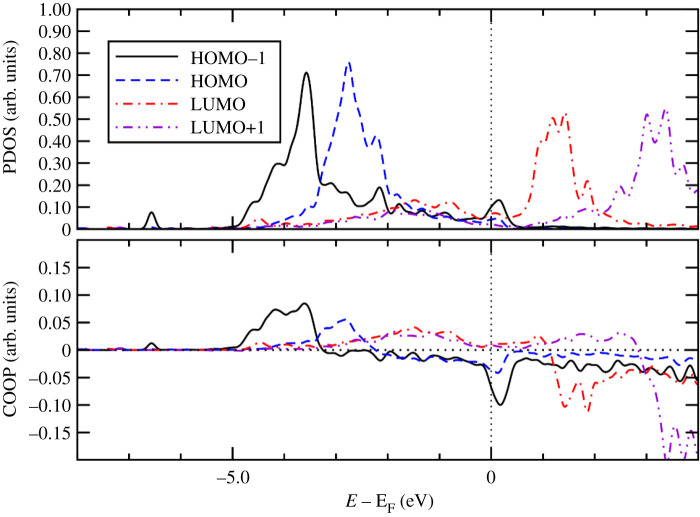
Density of states projected onto molecular orbitals (PDOS) and density of states weighted by crystal orbital overlap population (COOP) for the Ff molecule adsorbed on Pd(110). Gaussian broadening with the width of 0.01 eV was used. Origin of the energy is the Fermi level (E_F_).

**Figure 10.**
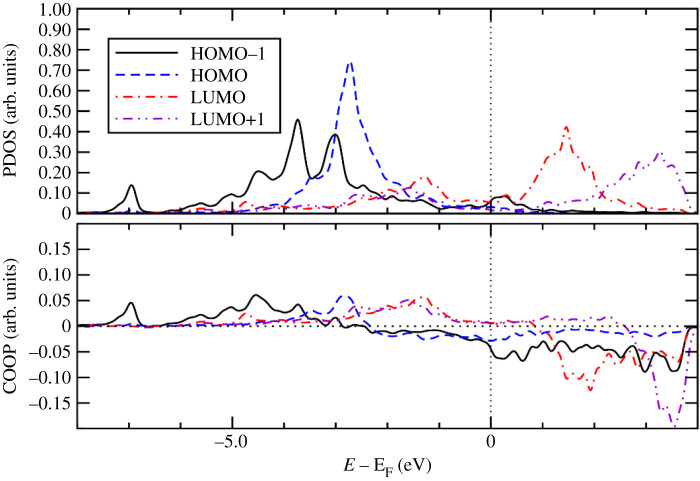
Density of states projected onto molecular orbitals (PDOS) and density of states weighted by crystal orbital overlap population (COOP) for the Ff molecule adsorbed on Pt(110). Gaussian broadening with the width of 0.01 eV was used. Origin of the energy is the Fermi level (E_F_).

**Figure 11.**
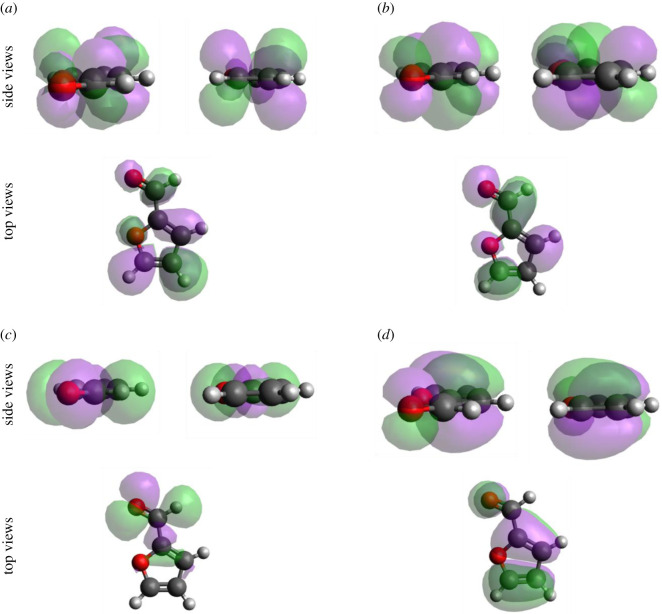
The wavefunctions for (*a*) LUMO+1, (*b*) LUMO, (*c*) HOMO and (*d*) HOMO-1 for the fully optimized furfural molecule.

To gain further insight into the bonding mechanism of the adsorption of the Ff molecule on the metal surfaces, we calculated PDOS onto molecular orbital and COOP using the STATE code. COOP characterizes the interaction between molecular orbital (MO) and the substrate states, and the positive COOP indicates the bonding interaction between MO and substrate states, whereas the negative COOP, an antibonding interaction. Figures 8–10, show PDOS and COOPs of the HOMO-1, HOMO, LUMO and LUMO+1 for the Ff molecule adsorbed on metal (110) surface. Overall, we can see significant hybridization of HOMO and HOMO-1 (figure 11) with the substrate states. At the same time, antibonding parts of the HOMO and HOMO-1 derived states (negative COOP for HOMO and HOMO-1) are partially occupied, indicating Pauli repulsion of HOMO and HOMO-1 with the substrate states. We see that the HOMO hybridizes significantly, and the degree of hybridization correlates with the adsorption strength. In the case of Pt(110), the HOMO derived states distribute broadly (to approx. 7.0 eV below the Fermi level). Furthermore, LUMO and LUMO-1 (figure 10) are partially occupied and hybridize with the substrate states as indicated by the corresponding COOPs, which contributes to the stabilization of the Ff molecule on the surface and is a factor determining the stability of the molecule.

## 4. Summary and conclusion

In this study, we reported that the Pt/TiO_2_ catalyst and Pd/TiO_2_ exhibited superior activity as compared to the Ni/TiO_2_ catalyst. Furthermore, it was observed that there is a difference not only in their activity but also in their selectivity profile. The Ni/TiO_2_ catalyst had a preferential selectivity towards THFA, whereas Pd/TiO_2_ and Pt/TiO_2_ favoured FA. To understand the fundamental nature of the interaction between the Ff molecule and the low index Ni, Pd and Pt surfaces, we performed DFT analyses and found that the adsorption energy of the Ff molecule is the largest on the Pt(111) surface, followed by those for the Ni(111) and the Pd(111) surface. On the (110) surfaces, the adsorption energy of the Ff molecule follows a similar trend to the (111) surfaces. However, on the M(110) surface, the adsorption energy of the Ff molecule is largest for the Ni surface, followed by those for the Pt and Pd surfaces. To understand better the probable reasons for the calculated trends of the adsorption energies we investigated the geometrical and the local electronic properties of the Ff/M(*h*,*k*,*l*) surfaces. The analyses of the geometrical properties show that depending on the metal surface the Ff molecule may adopt a different configuration, which may lead to different preferential hydrogenation reactions. The Bader charge analysis reveals electron transfer from metal to the Ff molecule on the Ni and Pd surfaces, while a reverse process occurs on the Pt surface, which is indicated by the presence of a residual negative charge on its exposed surface. From the density of states analysis, we conclude that the stability of the Ff molecule can be directly related to the metal *d-*states which hybridize with its nearest C and O *p*-orbitals. Based on our DFT analyses, we further conclude that the interactions between atoms with higher electronegativity and the metal *d-*states play a key role in determining the stabilities of the Ff/M(*h*,*k*,*l*) systems under investigation.

## Data Availability

All data are available from the Dryad Digital Repository: https://doi.org/10.5061/dryad.0zpc866zm [53].
